# Energy Renewal: Isothermal Utilization of Environmental Heat Energy with Asymmetric Structures

**DOI:** 10.3390/e23060665

**Published:** 2021-05-25

**Authors:** James Weifu Lee

**Affiliations:** Department of Chemistry and Biochemistry, Old Dominion University, Norfolk, VA 23529, USA; jwlee@odu.edu; Tel.: +1-757-683-4260

**Keywords:** thermotrophic function, energy renewal, asymmetric electron-gated function, isothermal electricity generation, transmembrane electrostatically localized protons, asymmetric membrane geometric effect on localized protons, negative entropy, isothermal environmental heat energy utilization

## Abstract

Through the research presented herein, it is quite clear that there are two thermodynamically distinct types (A and B) of energetic processes naturally occurring on Earth. Type A, such as glycolysis and the tricarboxylic acid cycle, apparently follows the second law well; Type B, as exemplified by the thermotrophic function with transmembrane electrostatically localized protons presented here, does not necessarily have to be constrained by the second law, owing to its special asymmetric function. This study now, for the first time, numerically shows that transmembrane electrostatic proton localization (Type-B process) represents a negative entropy event with a local protonic entropy change ($\Delta S_{L}$) in a range from −95 to −110 J/K∙mol. This explains the relationship between both the local protonic entropy change ($\Delta S_{L}$) and the mitochondrial environmental temperature (*T*) and the local protonic Gibbs free energy ($\Delta G_{L}=T\Delta S_{L}$) in isothermal environmental heat utilization. The energy efficiency for the utilization of total protonic Gibbs free energy ($\Delta G_{T}$ including $\Delta G_{L}=T\Delta S_{L}$) in driving the synthesis of ATP is estimated to be about 60%, indicating that a significant fraction of the environmental heat energy associated with the thermal motion kinetic energy (*k_B_T*) of transmembrane electrostatically localized protons is locked into the chemical form of energy in ATP molecules. Fundamentally, it is the combination of water as a protonic conductor, and thus the formation of protonic membrane capacitor, with asymmetric structures of mitochondrial membrane and cristae that makes this amazing thermotrophic feature possible. The discovery of energy Type-B processes has inspired an invention (WO 2019/136037 A1) for energy renewal through isothermal environmental heat energy utilization with an asymmetric electron-gated function to generate electricity, which has the potential to power electronic devices forever, including mobile phones and laptops. This invention, as an innovative Type-B mimic, may have many possible industrial applications and is likely to be transformative in energy science and technologies for sustainability on Earth.

## 1. Introduction

In the past four centuries, it was widely believed that environmental heat energy (the dissipated form of heat energy; also known as latent heat, or the temperature-dependent molecular thermal motion kinetic energy in the environment) could not be utilized unless there was a temperature gradient or difference. This is also one of the classic statements for the second law of thermodynamics [1,2,3]. Recently, through bioenergetics elucidation studies on the basis of a novel transmembrane electrostatic proton localization theory [4,5,6,7,8,9,10], it was surprisingly revealed that environmental heat energy can be isothermally utilized through “transmembrane electrostatically localized protons at a liquid–membrane interface” to help drive ATP synthesis in certain biological systems such as alkalophilic bacteria *Bacillus pseudofirmus* [11,12,13,14,15,16]. This finding indicated that proton-coupling bioenergetic systems may have a thermotrophic feature that can isothermally generate significant amounts of Gibbs free energy from environmental heat (dissipated-heat energy) through transmembrane electrostatically localized protons with asymmetric membrane structures [11,12,13,14,15,16,17]. Naturally, this raises an important scientific question: Can the second law of thermodynamics really be applied everywhere, including life systems such as the protonic bioenergetic system? We now have quite a clear answer to this fundamental question of paramount importance [13,14,15,16,18].

That is, for centuries, it was thought to be completely impossible to isothermally utilize the environmental heat energy dissipated in the ambient environment to do any useful work as stated by one of the classic second law statements [1,2,3]. That classic belief is still largely true for many systems and processes around us, including for the cars and computers that we use. However, we now know that in certain special biophysical molecular systems, such as in the system of transmembrane electrostatically localized protons [8,10,19] at the liquid–membrane interface [5,20,21], that this classic belief may not necessarily have to be always true [16]. 

It is generally understood that the second law still remains a very good law. However, it does not necessarily have to always be universal, as also implied by several independent studies [3,11,22,23,24,25,26,27,28,29,30,31,32]. For example, many biological processes, such as glycolysis, tricarboxylic acid cycle, and redox-driven proton transport, apparently well follow the second law. Meanwhile, we now also understand that the special protonic isothermal environmental heat energy utilization function as presented below in this article perfectly follows the first law (conservation of mass and energy) of thermodynamics, but is not necessarily constrained by the redox-driven proton pump system, which apparently well follows the second law of thermodynamics. 

Note that the second law of thermodynamics was developed from the Sadi Carnot cycle [33], which was based on the ideal gas law (*nRT* = *PV*; here *P* is pressure, *V* is volume and *n* is the number of moles), where the ideal molecular particles were assumed to have freedoms in a three-dimensional space (volume) without the consideration of asymmetric structures. In the case of protonic bioenergetic systems, the transmembrane electrostatically localized protons [4,5,7,8,9,10,12,16,34] are on a two-dimensional membrane surface with asymmetric properties, which is quite different from the assumed three-dimensional space (volume) system that the second law was based on. Therefore, one must be careful not to mindlessly apply something like the second law derived from a three-dimensional space (volume) system to a two-dimensional system without looking into the specific facts. Furthermore, the asymmetric biological membranes resulting from a billion years of natural evolution were not considered by the second law per se; this is another reason that one should be careful not to apply the second law mindlessly or blindly to certain special cases involving asymmetric systems without looking into the specifics. 

We now have at least two well-defined systems, mitochondria (present study) and alkalophilic bacteria (recent study) [16] with well-corroborated scientific evidence showing the special protonic isothermal environmental heat energy utilization that perfectly follows the first law (conservation of mass and energy) of thermodynamics but is not necessarily constrained by the redox-driven proton pump system that apparently well follows the second law of thermodynamics. As shown below in this article, the entropy change ($\Delta S_{L}$) for the localized proton-associated isothermal environmental heat utilization was indeed calculated to be a negative number. Therefore, this discovery, with the new understanding of the biological protonic isothermal environmental heat energy utilization, may represent a complementary development to the second law of thermodynamics and its applicability in bettering the science of protonic bioenergetics and energy renewal. 

This article presents a clear case for the thermotrophic function in mitochondria, where significant amounts of Gibbs free energy are acquired through the isothermal utilization of environmental heat energy with localized protons (protonic thermal motion kinetic energy) to perform useful work such as driving ATP synthesis in a way similar to that of the bacterial thermotrophic feature that Lee recently first identified in alkalophilic bacteria [16]. The research progress reported in this article may represent a breakthrough advance in the scientific field of entropy in relation to the applicability of the second law of thermodynamics, since it now is, for the first time, numerically demonstrated that the transmembrane electrostatic proton localization indeed represents a negative entropy event associated with the thermotrophic function. This study shows that there are two thermodynamically distinct types (A and B) of energetic processes naturally occurring on Earth; Type A energy processes follow the second law well, while Type B energy processes do not necessarily have to follow the second law because of their special asymmetric functions. More importantly, the discovery of the thermotrophic function may have profound scientific and practical implications for bettering the fundamental understanding of energy renewal [11] for sustainable development on Earth. Inspired by the discovery of the thermotrophic function, a new invention was made in the form of a series of methods for the creation and use of asymmetric function-gated isothermal electricity production systems for energy renewal by electrons *isothermally* utilizing environmental heat energy [30]. Therefore, this article also highlights the use of this invention in energy renewal for isothermal electricity generation (PCT International Patent Application Publication Number WO 2019/136037 A1). 

## 2. Discovery of Thermotrophic Function in Mitochondria

Through the following methods and biophysical chemistry analyses employing the transmembrane electrostatically localized protons theory (also called as the transmembrane electrostatic proton localization theory) [4,5,7,8,9,10,12,16,34], a novel mitochondrial thermotrophic phenomenon is now identified with well-corroborated evidence: isothermal utilization of environmental heat energy associated with the thermal motion kinetic energy (*k_B_T*) of transmembrane electrostatically localized protons in driving the synthesis of ATP. This thermotrophic function can lock a significant fraction of the environmental heat energy into ATP chemical energy. Remarkably, this finding is well corroborated by the structures and functions of mitochondrial cristae and corroborated by the asymmetric structures of mitochondrial respiratory-coupling sites, as highlighted below. 

## 3. Methods

### 3.1. Newly Formulated Protonic Motive Force Equation with Transmembrane Electrostatically Localized Protons

The basic process that leads to the synthesis of ATP in mitochondria involves creating an excess number of protons on one side of the mitochondrial inner membrane accompanied by a corresponding number of hydroxyl ions on the other side, for instance, through the respiratory electron-transport-coupled proton pumps across the mitochondrial inner membrane. According to the transmembrane electrostatic proton localization theory [4,5,7,8,9,10,12,16,34], the excess positively charged protons in an aqueous medium on one side of the mitochondrial inner membrane will electrostatically become localized at the liquid–membrane interface, attracting an equal number of excess negatively charged hydroxyl anions to the other side (matrix) of the mitochondrial inner membrane to form a “protons–membrane–anions capacitor structure”. This theory rests on the premise that a liquid water body acts as a protonic conductor, which is consistent with the well-established knowledge that protons quickly transfer among water molecules by the “hops and turns” mechanism first outlined by Grotthuss [35,36,37]. 

Therefore, a newly formulated equation for the protonic motive force (pmf) across a biological membrane considering the transmembrane electrostatically localized protons was introduced in the author’s most recent publications [8,10,16,34] as

(1) $$\mathrm{pmf}=\Delta \Psi +\frac{2.3\;RT}{F}\;\log_{10}\left(\left[H_{pB}^{+}\right]/\left[H_{nB}^{+}\right]\right)+\frac{2.3\;RT}{F}\;\log_{10}\left(1+\left[H_{L}^{+}\right]/\left[H_{pB}^{+}\right]\right)$$

Here, ΔΨ is the transmembrane electrical potential difference from the positive *p*-side to the negative *n*-side as defined by Mitchell [38,39], and Nicholls and Ferguson [40,41]; *R* is the gas constant; *T* is the absolute thermodynamic temperature; *F* is Faraday’s constant; $\left[H_{L}^{+}\right]$ is the concentration of transmembrane electrostatically localized protons at the liquid–membrane interface on the positive (*p*) side of the membrane; $\left[H_{pB}^{+}\right]$ is the proton concentration in the bulk aqueous *p*-phase (intermembrane space in the case of mitochondria); and $\left[H_{nB}^{+}\right]$ is the proton concentration in the bulk liquid *n*-phase (matrix in mitochondria). The first two terms of Equation (1) comprise the Mitchellian bulk phase-to-bulk phase proton electrochemical potential gradients [42,43,44] that we now refer to as the “classic” pmf; whereas the last term accounts for the “local” pmf from the transmembrane electrostatically localized protons at the liquid–membrane interface.

For an idealized protonic membrane capacitor, as previously reported [5,8,10,16,34,45], the concentration of the localized protons ${\left[H_{L}^{+}\right]}^{0}$ at the membrane–water interface on the positive (*p*) side is related to the transmembrane electrical potential difference ΔΨ by

(2) $${\left[H_{L}^{+}\right]}^{0}=\frac{C}{S}\cdot \;\frac{\Delta \Psi }{l\cdot F}\;$$

where C/S is the specific membrane capacitance per unit surface area, and l is the thickness of the localized proton layer. In actual mitochondria, non-proton cations in the aqueous media may exchange with protons localized at the liquid–membrane interface and thereby reduce their concentration. To account for this effect, as reported previously [5,8,10,16,34,45], we use

(3) $$\left[H_{L}^{+}\right]=\frac{{\left[H_{L}^{+}\right]}^{0}}{\prod_{i=1}^{n}\;\{K_{Pi}\left(\frac{\left[M_{pB}^{i+}\right]}{\left[H_{pB}^{+}\right]}\right)+1\}}\;$$

for the resulting concentration of transmembrane electrostatically localized protons $\left[H_{L}^{+}\right]$ at the liquid–membrane interface. Here, $\left[M_{pB}^{i+}\right]$ is the concentration of a non-proton cation such as Na^+^, K^+^, and Mg^2+^ in the bulk liquid *p*-phase and $K_{Pi}$ is the equilibrium constant for the cation to exchange with the localized protons.

It is noteworthy that all the physical quantities appearing in Equations (1)–(3) may, in principle, be determined through experimental measurements. There are no freely adjustable parameters. For the membrane and proton layer parameters in Equation (2), the calculations reported in this article have taken C/S = 13.2 mf/m^2^ as an averaged membrane capacitance based on measured experimental data [46] and l = 1 nm, which, as discussed in Refs. [5,8,10,16,21,34], is a reasonable thickness of the localized proton layer. It is also noteworthy that the thickness of the localized proton layer l is an important parameter that is not well known. It governs the magnitude of the localized proton concentration. Additionally, as discussed below, it may put constraints on the redox-driven electron-transport-coupled proton pumps and on the protonic user structures that are embedded within the mitochondrial inner membrane. 

Note that, in some of the literature, the membrane potential might have been reported as a “negative” number [47,48,49] due to using an opposite reference orientation (from *n*- to *p*-side) [50,51] in contrast to that of the ΔΨ defined in Equations (1)–(3) from the *p*-side to the *n*-side, as defined by Mitchell [38,39] and Nicholls and Ferguson [40,41]. In that case, special care must be taken to correct (remove) the negative sign for such membrane potential data with the opposite orientation, such as those in Refs. [48,52,53], before applying them to the pmf equations (Equations (1) and (2)) as previously reported [5,8,10,16,45]. 

### 3.2. The Relationship between Transmembrane Electrostatically Localized Protons and Local Protonic Gibbs Free Energy and Entropy Change

According to the transmembrane electrostatic proton localization theory [4,5,7,8,9,10,12,16,34], the amount of local pmf can be calculated using the following local pmf equation (which is consistent with the third term of Equation (1)):

(4) $$\mathrm{local}\;\mathrm{pmf}=\frac{2.3\;RT}{F}\;\log_{10}\left(1+\left[H_{L}^{+}\right]/\left[H_{pB}^{+}\right]\right)$$

Furthermore, protonic motive force (pmf) is equivalent to protonic Gibbs free energy ΔG according to a simple relation with the Faraday constant (F), as in the following equation:

(5) ΔG=−F  pmf 

Consequently, the amount of local protonic Gibbs free energy ($\Delta G_{L}$) resulting from transmembrane electrostatically localized protons can be calculated using the following equation:

(6) $$\Delta G_{L}=-2.3\;RT\;\log_{10}\left(1+\left[H_{L}^{+}\right]/[H_{pB}^{+}]\right)$$

We now understand that the ratio ($\left[H_{L}^{+}\right]/[H_{pB}^{+}]$) of the localized proton concentration $\left[H_{L}^{+}\right]$ at the membrane–liquid interface at the *p*-side to the bulk liquid phase proton concentration $\left[H_{pB}^{+}\right]$ at the same side in the mitochondria intermembrane space/crista space is related to the “negative entropy change” $\Delta S_{L}$, as shown in the following quantitative expression:

(7) $$\Delta S_{L}=-2.3\;R\;\log_{10}\left(1+\left[H_{L}^{+}\right]/[H_{pB}^{+}]\right)$$

In the study reported below, the localized proton bioenergetics analyses with Equations (1)–(7) are extended to mitochondria not only by making a better treatment for cation exchange using newly determined proton–cation exchange equilibrium constants [6,54] for sodium, potassium and magnesium cations, but also by incorporating this treatment to calculate the local protonic entropy change ($\Delta S_{L}$) and the total protonic Gibbs free energy change ($\Delta G_{T}$) including the classic protonic Gibbs free energy change ($\Delta G_{C}$) and the local protonic Gibbs free energy change ($\Delta G_{L}$) for a full range of membrane potentials $\left(\Delta ,\Psi \right)$ from 50 to 200 mV, including the measured values for ΔΨ, $\left[H_{pB}^{+}\right]$, and $\left[H_{nB}^{+}\right]$ from the well-documented animal mitochondria experimental study [52]. 

### 3.3. Published Data Utilized in Protonic Biophysical Chemistry Analyses

The thermotrophic function in mitochondria is presented herein through biophysical chemistry analyses with the transmembrane electrostatic proton localization theory [4,5,7,8,9,10,12,16,34], using published data from independent studies, including the experimental assay of mitochondrial ATP-ADP exchange by Chinopoulos et al. (2009) [52], where mitochondrial membrane potential ΔΨ was measured in a range from 60 to 160 mV. The experimental data of Chinopoulos et al. (2009) showed ATP synthesis, as measured by ATP efflux rate, at a membrane potential ΔΨ as low as anywhere between 60 and 80 mV (Figure 7C of Ref. [52]). More importantly, by measuring the matrix pH using pH-sensitive fluorescence ratio to the pH of the extracellular volume, their experimental work [52] showed that there is essentially no or little bulk-phase pH difference between the matrix and the intermembrane space across the mitochondrial inner membrane: the “∆pH_max_ is only ~0.11”. That is, under the given reaction medium pH 7.25 ($pH_{pB}$), mitochondria matrix pH during state three was about 7.35 ($pH_{nB}$). Another independent study [55] also consistently showed that the mitochondria matrix pH was about 7.3, which is essentially identical to that of the cytosol. These experimental observations are well corroborated by our latest experimental results from a biomimetic anode water–membrane–water cathode system, where the bulk-phase liquid pH in the anode liquid chamber remains about the same as that in the cathode liquid chamber before and after energization by excess protons at one side of the membrane and excess hydroxyl anions at the other side [6,11,21,54]. Therefore, the measured experimental parameters (data) of the reaction medium pH 7.25 (*pH_pB_*) and mitochondria matrix pH 7.35 (*pH_nB_*) during state three, as reported by Chinopoulos et al. (2009) [52], are used in the bioenergetics calculations here, as previously reported [8,10]. Accordingly, the total product of cation–proton exchange reduction factors, as shown in the denominator of Equation (3), is 1.29, which is close to 1, indicating a relatively minor role of cation–proton exchange at the liquid–membrane interface in modulating the transmembrane electrostatically localized proton concentration in mitochondria [8,10].

## 4. Results Showing Mitochondrial Thermotrophic Function 

### 4.1. Numerical Evidence for the Presence of Protonic Thermotrophic Function in Mitochondria

**Figure 1:**
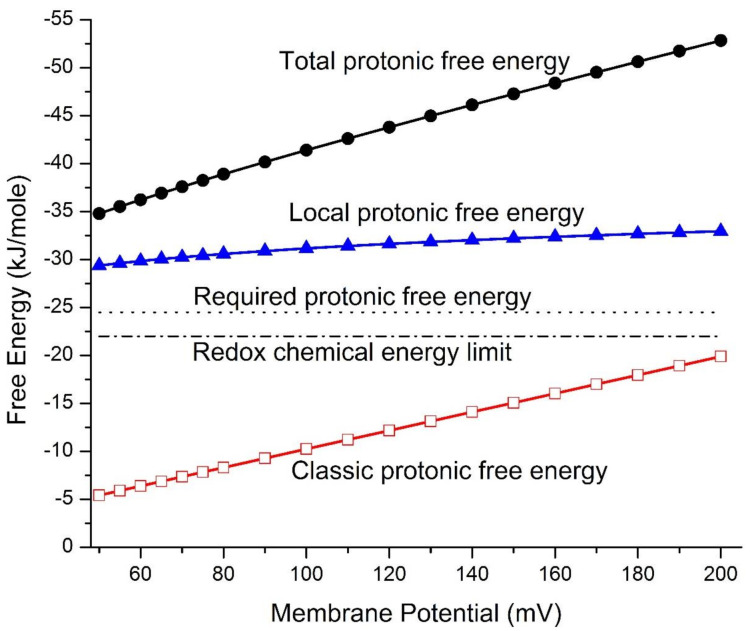
Gibbs free energy (Δ*G*) values including the total protonic Gibbs free energy ($\Delta G_{T}$), local protonic Gibbs free energy ($\Delta G_{L}$), and classic protonic Gibbs free energy ($\Delta G_{C}$) in mitochondria calculated as a function of membrane potential ΔΨ compared to the required protonic Gibbs free energy of --24.5 kJ/mol for ATP synthesis ($\Delta G_{Syn}$) and to the redox potential chemical energy upper limit ($\Delta G_{Chem}$) of --22.0 kJ/mol.

The mitochondrial protonic energetics properties, including mitochondrial protonic motive force (pmf)-associated Gibbs free energy changes (ΔG) and local protonic entropy changes ($\Delta S_{L}$), were calculated as a function of transmembrane electrical potential difference ΔΨ  using Equations (1)–(7) under the given reaction medium pH 7.25 ($pH_{pB}$), mitochondria matrix pH 7.35 ($pH_{nB}$), and taking cation–proton exchange into account as described in the methods above. As listed in Table 1 for a full range of mitochondrial membrane potentials (ΔΨ from 50 to 200 mV), the numerically calculated total protonic Gibbs free energy ($\Delta G_{T}$) is in the range from −34.9 to −52.9 kJ/mol, which includes both the classic protonic Gibbs free energy ($\Delta G_{C}$ from −5.42 to −19.9 kJ/mol) and the local protonic Gibbs free energy ($\Delta G_{L}$ from −29.5 to −33.1 kJ/mol). The calculated total protonic Gibbs free energy ($\Delta G_{T}$), in comparison to both the classic protonic Gibbs free energy ($\Delta G_{C}$) and the local protonic Gibbs free energy ($\Delta G_{L}$), is presented in Figure 1 as a function of the transmembrane potential difference ΔΨ for a full range of the membrane potentials (ΔΨ) from 50 to 200 mV. It is apparent from these results that the local protonic Gibbs free energy ($\Delta G_{L}$) from the transmembrane electrostatically localized protons dominantly contributes to the overall strength of the total protonic Gibbs free energy ($\Delta G_{T}$).

Note that the phosphorylation potential (ΔG_ATP_) required for ATP synthesis used by Slater [56] in his 1967 evaluation of the chemiosmotic hypothesis was +15.6 kcal/mol, which was measured by Cockrell et al. 1966 [57] in isolated rat liver mitochondria. Remarkably, this phosphorylation potential of +15.6 kcal/mol (equivalent to 65.3 kJ mol^−1^) for ATP synthesis is quite close to the magnitude of the critical free energy −63.5 kJ mol^−1^ for ATP hydrolysis in a functional animal heart cell obtained by Wu et al. 2008 [58], and thus it may be considered as a physiologically required phosphorylation potential (65.3 kJ mol^−1^) for ATP synthesis. According to this phosphorylation potential of +65.3 kJ mol^−1^, the physiologically required protonic Gibbs free energy ($\Delta G_{Syn}$) for ATP synthesis with a proton-to-ATP ratio of 8/3 in mitochondria should be −24.5 kJ mol^−1^ (−65.3 kJ mol^−1^/2.67). The proton-to-ATP ratio of 8/3 is consistent with the known structure of the animal mitochondrial F_0_F_1_-ATP synthase, which has three catalytic sites for ATP synthesis, driven by a flow of eight protons per revolution through the eight c-subunits in its nanometer-scale molecular turbine ring [59,60,61,62]. 

Based on the data in Table 1 and Figure 1, the classic protonic Gibbs free energy ($\Delta G_{C}$ from −5.42 to −19.9 kJ/mol) alone is not sufficient to explain the protonic energetics for ATP synthesis in mitochondria since the $\Delta G_{C}$ value is below the physiologically required protonic Gibbs free energy ($\Delta G_{Syn}$) of −24.5 kJ mol^−1^ for ATP synthesis at any point in a full range of mitochondrial membrane potentials (ΔΨ) from 50 to 200 mV. The in vivo mitochondrial membrane potential (ΔΨ) [63,64,65] values that have been experimentally observed were mostly below 150 mV, including 56 mV, 105 ± 0.9 mV, and 81 ± 0.7 mV reported by Zhang et al. (2001) [53] and 91 ± 11 mV and 81 ± 13 mV measured by Gurm et al. (2012) using the techniques of 4-[^18^F]fluorophenyltriphenylphosphonium and in vivo positron emission tomography (PET) measurement [66], as well as 114 mV and 123 mV measured in swine and human, respectively, using an improved PET-based method by Alpert et al. 2018 [63] and by Pelletier-Galarneau et al. 2020 [67]. We now understand that the classic Mitchellian chemiosmotic theory [42,43,44] cannot explain the mitochondrial energetics in living cells because it fatally misses to account for the local protonic Gibbs free energy contribution from the transmembrane electrostatically localized protons at the liquid–membrane interface in mitochondria [8,10,16]. 

These findings are well corroborated by the mysterious problem previously noticed by Silverstein (2014) as a “thermodynamic efficiency of 113%” in mitochondria at a membrane potential of around 80 mV [68]. According to the classic Mitchellian pmf equation [42,69,70], to avoid the “impossibly high efficiency (>100%)” for mitochondria, one would have to “adjust” the bulk-phase “ΔpH (in-out)” to an arbitrary value of at least “+2.5”. However, it is now quite clear that the bulk-phase ΔpH (in-out) is nearly zero: “ΔpHmax is only ~0.11” based on the modern experimental measurements [52] and modeling analysis of mitochondria [71]. The observed bulk-phase ΔpH of nearly zero in mitochondria [52] is also corroborated by the prediction from the transmembrane electrostatic proton localization model [5,8,10,11,12] with the understanding that mitochondrial inner membrane is rather impermeable to ions [72,73]. Another independent study using a pH-sensitive GFP [74] has also now shown “that the intracristae lumen does not provide a reservoir for substrate protons for ATP synthesis” indicating “kinetic coupling of the respiratory chain with ATP synthase, but not proton gradients, drives ATP production in cristae membranes”. Therefore, there is really no way for the classic Mitchellian chemiosmotic theory [42,43,44] alone to explain the energetics in mitochondria; there must be another rather disparate protonic energetics mechanism in driving the synthesis of ATP through the F_0_F_1_-ATP synthase. 

We now understand that this disparate protonic energetics mechanism acts through the local protonic Gibbs free energy ($\Delta G_{L}$) from the transmembrane electrostatically localized protons based on the transmembrane electrostatic proton localization theory [4,5,7,8,9,10,12,16]. As shown in Table 1 and Figure 1, the local protonic Gibbs free energy ($\Delta G_{L}$) was calculated to be in a range from −29.5 to −33.1 kJ mol^−1^, whereas the classic protonic Gibbs free energy ($\Delta G_{C}$) is in the range from −5.42 to −19.9 kJ mol^−1^ at a range of membrane potential (ΔΨ) from 50 to 200 mV. The total protonic Gibbs free energy ($\Delta G_{T}$), which is the sum of the classic protonic Gibbs free energy ($\Delta G_{C}$) and the local protonic Gibbs free energy ($\Delta G_{L}$), is in the range from −34.9 to −52.9 kJ mol^−1^. All of these $\Delta G_{L}$ and $\Delta G_{T}$ values (Table 1) are well above the physiologically required $\Delta G_{Syn}$ of −24.5 kJ mol^−1^ for ATP synthesis at any of the membrane potential (ΔΨ) values in the range from 50 to 200 mV. Thus, the newly formulated set of protonic Gibbs free energy equations (Equations (1)–(6)) consistently provides an excellent elucidation for the energetics in mitochondria without requiring any arbitrary adjustment in the number of the bulk-phase “ΔpH (in-out)” that the previous study [68] required.

**Figure 2:**
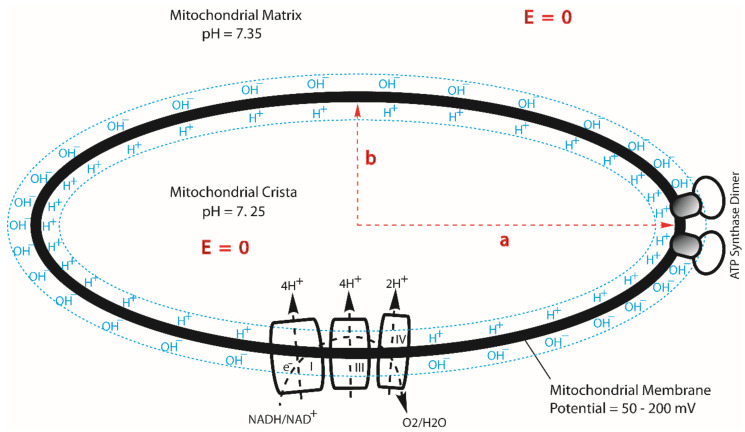
The geometric effect of cristae in enhancing the density of transmembrane electrostatically localized protons at the cristae tips where the F_0_F_1_-ATP synthase enzymes are located, as illustrated in a cross-section for an ellipsoidal-shaped mitochondrial crista: transmembrane electrostatic proton localization (protonic capacitor) model illustrating how excess protons (H^+^) and hydroxyl ions (OH^–^) could be electrostatically localized at the water–membrane interfaces along the two sides of the mitochondrial inner membrane before proton–cation exchange as it would be in a theoretically pure water–membrane–water system. Note that this cross-section can be considered as a special result from the tri-axial (a, b, and c) protonic conducting ellipsoidal crista equation in three-dimensional x, y, and z coordinates for its middle cross-section (where the z coordinate is zero). Adapted from Ref. [10].

The redox potential chemical energy upper limit ($\Delta G_{Chem}$) that could be supported by the entire respiratory redox-driven proton pump system in mitochondria was calculated to be about −22.0 kJ mol^−1^ as follows. The redox potential difference between the electron donor NADH (E_m,7_ = −320 mV) to the terminal electron acceptor O_2_ (E_m,7_ = +820 mV) in this system is known to be about 1140 mV [40]. For each pair of electrons from NADH to pass through the respiratory chain (complexes I, III and IV) to the terminal electron acceptor O_2_ as shown in Figure 2, the system drives the translocation of 10 protons across the membrane from the matrix to the intermembrane space/crista space [10]. That is, it couples the translocation of 5 protons per electron across the membrane. Therefore, the maximum pmf that could be generated by the redox-driven proton pump system would be about 228 mV per proton (1140 mV/5 protons), which is equivalent to −22.0 kJ mol^−1^ (= −F  228 mV) as the redox potential chemical energy limit ($\Delta G_{Chem}$). 

Note that even the redox potential chemical energy limit $\Delta G_{Chem}$, which represents the theoretical chemical energy upper limit (−22.0 kJ mol^−1^) in the classic Mitchellian bulk phase-to-bulk phase proton electrochemical potential gradients [42,43,44], is still below the physiologically required $\Delta G_{Syn}$ of −24.5 kJ mol^−1^ for ATP synthesis in mitochondria. Therefore, the known classic chemical energy process alone is not sufficient to explain the protonic bioenergetics. This also indicates that there must be another fundamentally disparate biophysical energetics mechanism in mitochondria, which we now know to be the thermotrophic function, isothermally using environmental heat energy associated with transmembrane electrostatically localized protons at the liquid–membrane interface in driving the synthesis of ATP molecules.

The newly calculated total protonic Gibbs free energy ($\Delta G_{T}$) data, including the local protonic Gibbs free energy ($\Delta G_{L}$) (Table 1 and Figure 1), showed plenty of protonic Gibbs free energy, well above the $\Delta G_{Syn}$ of −24.5 kJ mol^−1^, for ATP synthesis through the oxidative phosphorylation by mitochondrial F_0_F_1_-ATP synthase even at a relatively low membrane potential (ΔΨ) level anywhere in the full range from about 50 mV to 200 mV. This finding is remarkably in line with the independent observations of mitochondrial membrane potentials (ΔΨ) in living cells being mostly about 56 mV, 105 ± 0.9 mV and 81 ± 0.7 mV [53], 91 ± 11 mV and 81 ± 13 mV [66], and also 114 mV [63] and 123 mV [67] where apparently significant amounts of ATP are synthesized at such relatively low mitochondrial membrane potentials to support the growth and activities of the living cells. 

### 4.2. Transmembrane Electrostatically Localized Protons with Asymmetric Membrane Structures Isothermally Using Environmental Heat to Synthesize ATP

It is encouraging that the total protonic Gibbs free energy ($\Delta G_{T}$) values calculated as a function of the membrane potential (ΔΨ) in a range from 50 mV to 200 mV from the use of Equations (1)–(6) for mitochondria are all larger than the $\Delta G_{Syn}$ of −24.5 kJ mol^−1^ for synthesizing ATP. However, it was a surprising result that the total protonic Gibbs free energy ($\Delta G_{T}$) values in the whole range from −34.9 to −52.9 kJ mol^−1^, as shown in Table 1 and Figure 1, are significantly larger than the redox potential chemical energy limit $\Delta G_{Chem}$ (−22.0 kJ mol^−1^) that could be maximally supported by the redox-driven proton pump system. 

How is it possible that the total transmembrane protonic Gibbs free energy ($\Delta G_{T}$) exceeds the redox potential chemical energy upper limit $\Delta G_{Chem}$ (−22.0 kJ mol^−1^) imposed by the respiratory redox-driven proton pump system? We now understand, here, that the local protonic Gibbs free energy ($\Delta G_{L}$) from the transmembrane electrostatically localized protons is not constrained by the chemical energy limit $\Delta G_{Chem}$ of the respiratory redox-driven proton pump system and that the environmental heat energy, which is also known as the temperature-dependent molecular thermal motion kinetic energy, is effectively used by the transmembrane electrostatically localized protons for ATP synthesis (driving the synthesis of ATP from ADP and Pi through F_0_F_1_-ATP synthase), for at least one of the following four reasons.

First, the geometric effect of mitochondrial cristae can enhance the density of transmembrane electrostatically localized protons at the cristae tips [10], where the F_0_F_1_-ATP synthase enzymes are located (Figure 2), by a factor of more than 10. As recently reported [10], the ratio of the localized proton concentration at the crista tip (${\left[H_{L}^{+}\right]}^{0}{}_{tip}$) to that at the crista flat region (${\left[H_{L}^{+}\right]}^{0}{}_{flat}$) is equal to the axial ratio (a/b) of an ellipsoidal mitochondrial crista. Consequently, for an ellipsoidal crista with a length of 200 nm and width of 20 nm, the localized proton concentration at the crista tip (${\left[H_{L}^{+}\right]}^{0}{}_{tip}$) can be as high as 10 times that at the flat region (${\left[H_{L}^{+}\right]}^{0}{}_{flat}$). This translates to a localized proton-associated liquid–membrane interface pH difference of about one pH unit between the crista tip (ridge) and the flat region within the same crista. That is, the localized proton-associated pH ($-Log_{10}({\left[H_{L}^{+}\right]}^{0}{}_{tip})$) at a crista tip (or ridge) can be significantly lower (by as much as “−1.00” pH unit) than that ($-Log_{10}({\left[H_{L}^{+}\right]}^{0}{}_{flat})$) at the flat region within the same crista. It is now known that the proton-pumping “respiratory supercomplexes” (complexes I, III and IV) are situated at the relatively flat membrane regions where the localized proton concentration (${\left[H_{L}^{+}\right]}^{0}{}_{flat}$) is relatively lower whereas the ATP synthase dimer rows are located at the cristae ridges (tips) where the transmembrane electrostatically localized proton concentration (${\left[H_{L}^{+}\right]}^{0}{}_{tip}$) is significantly higher as shown in Figure 2 [10,75,76,77,78,79,80]. Consequently, even if the protonic outlets of the complexes I, III and IV are somehow in contact with the transmembrane electrostatically localized proton layer at the crista flat region so that their activities would be equilibrated with the redox potential chemical energy limit $\Delta G_{Chem}$ (−22.0 kJ mol^−1^), the total protonic Gibbs free energy ($\Delta G_{T}$) at the crista tip can still be as high as −27.9 kJ mol^−1^ (= −22.0 + (−5.89)) since the transmembrane electrostatically localized proton density at the crista tip can be as high as 10 times that of the crista flat region (i.e., enhanced by “−1.00” pH unit), equivalent to an additional effective protonic Gibbs free energy of −5.89 kJ mol^−1^ owing to the crista geometric effect on the transmembrane electrostatically localized protons at the liquid–membrane interface [10]. 

Second, the transmembrane electrostatically localized protons are dynamically mobile on the two-dimensional membrane surface, but not free to move away from the liquid–membrane interface, since they are transmembrane electrostatically held at the membrane surface [8,11,21]. This transmembrane electrostatic attracting force across a biomembrane with its thickness of about 4 nm and dielectric constant (κ) of about 3 units has recently been calculated to be as high as 1.92 × 10^−11^ Newtons (N) [34]. To physically move a localized proton away from the liquid–membrane interface at the membrane surface by a nanometer (1 nm) towards the bulk liquid phase would require a work (W) of about 1.92 × 10^−20^ Joules (1.92 × 10^−11^ N × 1.0 × 10^−9^ m), which is 4.5 times as much as the Boltzmann k_B_T thermal kinetic energy (4.28 × 10^−21^ joule) at a body temperature of 37 °C (310 K). Therefore, the localized protons/cations–membrane–anions capacitor system can be quite stable under physiological temperature conditions [34]. Consequently, the dynamic thermal motion (environmental heat) of the transmembrane electrostatically localized protons at the liquid–membrane interface will cause certain protons to enter the protonic opening (F_0_ proton channel inlet) of the ATP synthase and be used to drive its nanometer-scale molecular turbine for synthesis of ATP from ADP and Pi [61,81,82].

Third, the transmembrane electrostatically localized protons may not be directly coupled to the membrane-embedded redox proton pumps, namely, the respiratory chain complexes I, III, and IV [83]. If they were, their ability to do work would be constrained by the energetics of the oxidative-respiratory electron-transport-coupled proton pumping system. A natural explanation of why this does not occur would be that the exit points for the translocated protons are outside of the surface layer of the transmembrane electrostatically localized protons. 

Fourth, to effectively make use of the transmembrane electrostatically localized proton thermal motion kinetic energy, the protonic entry point for the ATP synthase should be inside the localized proton surface layer. In this way, the redox-driven protonic pump activity interacts with the protonic activity in the bulk liquid phases but not with that of the localized protonic layer at the liquid–membrane interface. Only the transmembrane electrical potential difference and the bulk liquid-phase protonic activity at the two sides of the membrane equilibrate with the proton-pumping respiratory electron-transport-chain activity. Consequently, the localized protonic thermal motion kinetic energy provides additional free energy that may be used isothermally by the F_0_F_1_-ATP synthase and is not limited by the redox potential chemical energy limit $\Delta G_{Chem}$ of −22.0 kJ mol^−1^. 

**Figure 3:**
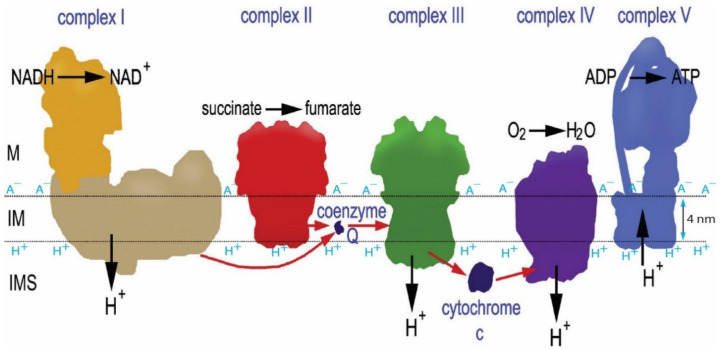
The known structures of mitochondrial respiratory membrane protein complexes I, II, III, IV, and F_0_F_1_-ATP synthase (complex V) in relation to the location of the membrane surfaces indicated by the horizontal dotted lines. The thickness of the membrane lipid bilayer (in between two dotted lines) is known to be about 4 nm, with reference to which the protonic outlets of the proton pumping complexes I, III and IV can be seen to protrude by about 1–3 nm into the bulk liquid phase, while the protonic inlet of F_0_F_1_-ATP synthase (complex V) is located at the transmembrane electrostatically localized proton layer along the membrane surface. Adapted from Ref. [16], which was adapted and modified from a schematic representation of the oxidative-respiratory phosphorylation system given in Ref. [84].

Remarkably, the known structures of the mitochondrial respiratory protein complexes, as determined by cryo-electron microscopy and other molecular structural studies [75,76,77,83,84,85,86,87,88], fit amazingly well with this explanation. As shown in Figure 3, every one of the mitochondrial respiratory proton-pumping protein complexes I, III, and IV protonic outlets do indeed protrude away from the membrane surface by about 1–3 nm into the bulk liquid *p*-phase (intermembrane space, IMS), while the protonic inlet of the ATP synthase (complex V) is located right at the localized proton layer along the membrane surface. Note that complex II, which does not pump protons and thus does not need to protrude, indeed does not protrude away from the membrane surface at the IMS side, as the author also predicted.

Apparently, a billion years of natural evolution has already made the asymmetric structures of the mitochondrial membrane (Figure 3) perfect for constituting this amazing isothermal environmental heat use function with transmembrane electrostatically localized protons. This is also, remarkably, corroborated by our experimental observations that the localized protons at the liquid–membrane interface are “not detectable to a pH electrode in the bulk liquid phase” [6,11,21,54]. The mitochondrial systems apparently take advantage of this feature by “purposely” putting the protonic outlets of their respiratory proton-pumping protein complexes I, III and IV into the bulk liquid phase to avoid contact with transmembrane electrostatically localized protons at the liquid–membrane interface while keeping the protonic mouth of the F_0_F_1_-ATP synthase precisely at the membrane surface to effectively use the localized protons that dominantly contribute to the total protonic Gibbs free energy ($\Delta G_{T}$), as shown in the data presented in Table 1 and Figure 1.

Therefore, we can now start to understand that the transmembrane electrostatically localized protons in combination with the asymmetric structural features of mitochondrial membrane especially regarding the positions of the proton pump outlets and the mouth of the localized proton users such as F_0_F_1_-ATP synthase with respect to the localized proton layer along the *p*-side of the membrane may constitute this special isothermal environmental heat use function. No new energy is created or destroyed here. Therefore, this special isothermal environmental heat use function perfectly follows the first law (conservation of mass and energy) of thermodynamics, but is not necessarily constrained by the redox-driven proton pump system that apparently well follows the second law of thermodynamics. 

Fundamentally, it is the transmembrane electrostatic proton localization with the effect of water as a protonic conductor that enables the formation of a localized excess proton layer at the liquid–membrane interface over the mouths of the protonic energy users including the F_0_F_1_-ATP synthase (Figure 3). The formation of a localized excess proton layer at the water–membrane interface apparently results in some kind of “negative entropy effect” [11,12,16] that brings the excess protons to the mouths of the protonic energy users, where the protons can isothermally utilize their molecular thermal motions (proton thermal kinetic energy *k_B_T*) possibly including their Brownian motion to push through the doors of F_0_F_1_-ATP synthase in driving ATP synthesis. Note, in the third term of Equation (1) for the local pmf, the utilization of proton thermal kinetic energy *k_B_T* is expressed as *RT* (= *k_B_T* · *N*_A_), which is equal to the product of the Boltzmann constant *k_B_*, the mitochondrial environmental temperature *T* and the Avogadro constant *N*_A_.

For the delocalized protons in the bulk liquid phase that are far away from the membrane surface, their thermal motions are not within the striking distance for them to hit into the F_0_F_1_-ATP synthase protonic channel to drive the rotary molecular machinery for ATP synthesis. Theoretically, in some extent, the delocalized protons could also do the work when they are at the liquid–membrane interface near the F_0_F_1_-ATP synthase. Therefore, the thermal energy factor *RT* (= *k_B_T* · *N*_A_) is also in the second term of Equation (1). However, the value of $\log_{10}\left(\left[H_{pB}^{+}\right]/\left[H_{nB}^{+}\right]\right)$ was nearly zero in this case; consequently, the delocalized protons in mitochondria did not significantly contribute to the isothermal utilization of environmental heat in driving ATP synthesis here. 

Notably, the isothermal utilization of dissipated-heat energy (environmental heat) by electrostatically localized protons at the mitochondrial liquid–membrane interface occurs without any physical phase transition such as the conventional liquid–gas phase change that would be required in the classic latent heat of water vaporization. Both the mitochondrial matrix liquid *n*-phase and the inter-membrane-space liquid *p*-phase essentially stay the same during the isothermal utilization of environmental heat through electrostatically localized protons. Furthermore, there is no temperature difference across the mitochondrial membrane. Therefore, the utilization of dissipated-heat energy from the mitochondrial temperature environment through transmembrane electrostatically localized protons at the mitochondrial liquid–membrane interface in driving ATP synthesis discovered here indeed represents a novel isothermal energy renewal event without any phase transition.

The proton-coupling bioenergetics systems operate widely in nearly all organisms known today. Therefore, through the present study, as presented above, it is now quite clear that this special biological isothermal environmental heat utilization process associated with transmembrane electrostatically localized protons has probably already occurred for billions of years on Earth. As shown in Figure 1, the amount of local protonic Gibbs free energy as calculated according to Equations (1)–(6) quantitatively represents the activity of this amazing isothermal environmental heat utilization function with localized protons. That is, the local protonic Gibbs free energy ($\Delta G_{L}$) equation (Equation (6)) has fundamental scientific significance in relation to isothermal environmental heat energy utilization with transmembrane electrostatically localized protons. Accordingly, it is the ratio ($\left[H_{L}^{+}\right]/[H_{pB}^{+}]$) of the localized proton concentration $\left[H_{L}^{+}\right]$ at the liquid–membrane interface to the bulk liquid-phase proton concentration $\left[H_{pB}^{+}\right]$ in the intermembrane space/crista space at the same *p*-side of the mitochondrial membrane that is mathematically related to this special thermotrophic feature, which is quite surprising.

### 4.3. Negative Entropy Changes for Protonic Gibbs Free Energy with Isothermal Environmental Heat Utilization

Based on the transmembrane electrostatic proton localization theory [4,5,7,8,9,10,12,16], when the excess positive charges (protons/cations) reach the liquid–membrane interface, they attract the excess negative charges (anions) at the other side of the membrane (Figure 2 and Figure 3). Consequently, the protons that are electrostatically localized in this way are not free to move away from the membrane surface because of the transmembrane electrostatic attraction force (1.92 × 10^−11^ N per proton–membrane–anion pair) between the excess positive charges (protons) at one side of the membrane and the excess negative charges (anions) at the other side of the membrane, although they can move freely within the liquid–membrane interface along the membrane surface in a quick/dynamic manner. This results in the formation of a protonic membrane capacitor (as shown Figure 2 and Figure 3) that can be quite stable under physiological temperature conditions, as was well corroborated by our recent experimental demonstration of a protonic capacitor, showing the formation of a transmembrane electrostatically localized layer of excess protons at the water–membrane interface in biomimetic experiments [6,21]. We now understand that the formation of such a localized protons–mitochondrial inner membrane–anions capacitor system represents some kind of “negative entropy event” [11,12,16] that brings the excess protons to the mouths of the protonic users, where the protons can isothermally utilize their molecular thermal motions (proton thermal kinetic energy *k_B_T*), possibly including their Brownian motion to push through the doors of F_0_F_1_-ATP synthase in driving ATP synthesis as mentioned above.

Here, we will provide numerical evidence for this protonic negative entropy change event by analyzing the local protonic entropy change ($S_{L}$) using Equation (7). Note, as shown previously, the protonic motive force (pmf) is equivalent to Gibbs free energy G according to a simple relation with the Faraday constant (F), as shown in Equations (5) and (6). Consequently, the amount of local protonic Gibbs free energy ($G_{L}$) resulting from this special isothermal environmental heat utilization function with transmembrane electrostatically localized protons can be calculated using Equation (6). As we now understand, the ratio ($\left[H_{L}^{+}\right]/[H_{pB}^{+}]$) of the localized proton concentration $\left[H_{L}^{+}\right]$ at the membrane–liquid interface at the *p*-side to the bulk liquid-phase proton concentration $\left[H_{pB}^{+}\right]$ at the same side in the intermembrane space/crista space is related to the “negative entropy change” $\Delta S_{L}$, as shown in the quantitative expression in Equation (7).

According to this local protonic entropy ($\Delta S_{L}$) equation (Equation (7)), as long as the transmembrane electrostatically localized proton concentration $\left[H_{L}^{+}\right]$ is above zero, the entropy change ($\Delta S_{L}$) is mathematically shown here as a negative number. That is, the entropy change for the isothermal environmental heat utilization process is indeed negative as long as the localized proton concentration $\left[H_{L}^{+}\right]$ is above zero in mitochondria. 

As listed in Table 1, the entropy change ($\Delta S_{L}$) calculated from Equation (7) for the localized proton-associated isothermal environmental heat utilization process is in the range from −95.1 to −107 joules per Kelvin per mole (J/K·mol) when the localized proton concentration $\left[H_{L}^{+}\right]$ at the liquid–membrane interface is in the range from 5.30 to 21.2 mM, which is a function of the membrane potential (ΔΨ) in a range from 50 to 200 mV. This is an important result (Table 1), since it is now, for the first time, numerically shown that the transmembrane electrostatic proton localization/protonic membrane capacitor formation does indeed represent a negative entropy event. 

Consequently, the amount of local protonic Gibbs free energy $\Delta G_{L}$ created from the utilization of environmental heat energy through the localized protons is the product (*T*$\Delta S_{L}$) of the local protonic entropy change $\Delta S_{L}$ and the mitochondrial thermodynamic environmental temperature *T* as shown in the following equation:

(8) $$\Delta G_{L}=T\Delta S_{L}$$

As shown in the data presented in Table 1, the local protonic Gibbs free energy ($\Delta G_{L}$) values as calculated with Equation (8) (equivalent to Equation (6)) are in the range from −29.5 to −33.1 kJ/mol, where the transmembrane electrostatically localized proton concentration $\left[H_{L}^{+}\right]$ at the liquid–membrane interface is in the range from 5.30 to 21.2 mM, which is a function of the membrane potential (ΔΨ) in a range from 50 to 200 mV. The so-created local Gibbs free energy ($\Delta G_{L}$ from −29.5 to −33.1 kJ/mol) is additive to the classic protonic Gibbs free energy ($\Delta G_{C}$ from −5.42 to −19.9 kJ/mol), resulting in the total protonic Gibbs free energy ($\Delta G_{T}$ from −34.9 to −52.9 kJ/mol). This explains the relationship between local protonic entropy change ($\Delta S_{L}$) and mitochondrial environmental temperature (*T*) and the associated local protonic Gibbs free energy ($\Delta G_{L}$ $=T\Delta S_{L}$) in isothermal environmental heat utilization. It numerically shows the negative entropy event with protonic membrane capacitor formation in relation to isothermal environmental heat utilization, which may fundamentally represent a novel understanding in energetics that has not been fully recognized before.

### 4.4. Protonic Gibbs Free Energy Utilization Efficiency in Relation to Thermotrophic Energetics

From the data (Table 1) of the protonic Gibbs free energy (Δ*G*) values calculated as a function of transmembrane potential ΔΨ compared to the physiologically required $\Delta G_{Syn}$ of −24.5 kJ mol^−1^ for ATP synthesis and to the redox potential chemical energy limit $\Delta G_{Chem}$ of −22.0 kJ/mol as shown Figure 1, we now understand that large amounts of local protonic Gibbs free energy ($\Delta G_{L}$) are formed through isothermally utilizing the environmental heat energy from the human body environment by the localized protons in mitochondria. The ratio of local protonic Gibbs free energy ($\Delta G_{L}$) to classic protonic Gibbs free energy ($\Delta G_{C}$) is in the range from 5.44 to 1.66, depending on the membrane potentials in the range from 50 to 200 mV, respectively. This result indicates that the amounts of local protonic Gibbs free energy ($\Delta G_{L}$) created through isothermal utilization of environmental heat energy by the localized protons are much greater than the classic protonic free energy ($\Delta G_{C}$). 

For example, at an in vivo membrane potential of 120 mV, the ratio of local protonic free energy ($\Delta G_{L}$) to classic protonic free energy ($\Delta G_{C}$) is 2.61:1. That is, mitochondria obtain 72% (2.61/3.61 = 0.72) of the total Gibbs free energy ($\Delta G_{T}\;$= −43.9 kJ/mol) from the localized proton-associated isothermal utilization of environmental heat energy ($\Delta G_{L}$). Therefore, it is now quite clear that mitochondria-powered organisms, including humans, have a significant thermotrophic property, in addition to being chemotrophs.

As reported above (Table 1), the entropy change ($\Delta S_{L}$) calculated from Equation (7) for the localized proton-associated isothermal environmental heat utilization is in a range between −95.1 and −107 J/K·mol. This is an important result, since it now, for the first time, numerically demonstrates that the transmembrane electrostatically proton localization indeed represents a negative entropy event, as mentioned above. Therefore, our discovery with the new understanding on the protonic thermotrophic function (Equations (1)–(8), Table 1, and Figure 1, Figure 2 and Figure 3) may represent a complementary development to the second law of thermodynamics and its applicability in bettering the science of protonic bioenergetics. 

When the transmembrane electrostatically localized protons utilize environmental heat energy (*k_B_T*) in helping drive the synthesis of ATP from ADP and Pi through the F_0_F_1_-ATP synthase (Figure 2 and Figure 3), a fraction of the environmental heat (*k_B_T*) energy may be locked into the chemical form of energy in ATP molecules, thus theoretically resulting in a small drop in the environmental temperature because of the localized proton-associated isothermal environmental heat utilization. However, in mitochondria and cells, there are many other processes (including the glycolysis, tricarboxylic acid cycle, and the redox-driven proton-pumping electron transport activities, as well as ATP utilization processes such as ATP hydrolysis) releasing heat energy, which could mask the isothermal environmental heat energy utilization process. That is, the energetic phenomenon of mitochondria (and the cells) may represent a complicated mixture of the chemotrophic processes and the isothermal environmental heat energy utilization process. This could probably also explain why it took so long for human beings on Earth to finally figure this out here.

Based on the physiologically phosphorylation potential of +65.3 kJ mol^−1^ required for ATP synthesis, the energy efficiency for the utilization of total protonic Gibbs free energy ($\Delta G_{T}$) including local protonic Gibbs free energy ($\Delta G_{L}$) in driving the synthesis of ATP can now be estimated. For example, according to the data in Table 1, at a mitochondrial membrane potential of 100 mV, where the total protonic Gibbs free energy ($\Delta G_{T}$) is −41.5 kJ mol^−1^ (including the $\Delta G_{L}$ of −31.3 kJ mol^−1^), the energy efficiency for the utilization of total protonic Gibbs free energy ($\Delta G_{T}$) in driving the synthesis of ATP is now estimated to be about 60% (65.3 × 100%/(2.67 × 41.5)), which thermodynamically appears to be a quite reasonable energy conversion efficiency. This also indicates that a significant portion of the total protonic Gibbs free energy ($\Delta G_{T}$) including the local protonic free energy ($\Delta G_{L}$) from the effect of transmembrane electrostatically localized protons associated with the isothermal utilization of the environmental heat thermal motion kinetic energy (k_B_T) can indeed be locked to the ATP chemical energy.

## 5. Thermotrophy-Inspired Invention for Isothermal Electricity Generation

Inspired by the discovery of the thermotrophic function of isothermally utilizing environmental heat energy through transmembrane electrostatically localized protons with asymmetric structures to do useful work such as driving ATP synthesis, a new invention (PCT International Patent Application Publication Number WO 2019/136037 A1) was developed, providing a series of methods for the creation and use of asymmetric function-gated isothermal electricity production systems for energy renewal with electrons isothermally utilizing environmental heat energy [30]. 

**Figure 4:**
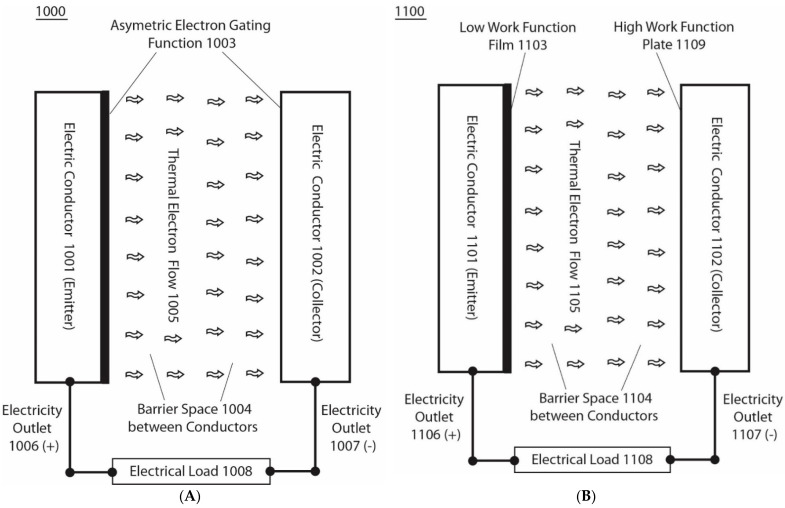
(**A**) Asymmetric function-gated isothermal electron power generator system 1000 comprising an asymmetric electron-gating function across a membrane-like barrier space that separates two electric conductors. (**B**) Basic unit of an asymmetric function-gated isothermal electron power generator system 1100, comprising a barrier space such as a vacuum space that separates a pair of electric conductors: one of them has a low-work-function film to act as a thermal electron emitter and the other has a high-work-function plate surface to serve as an electron collector.

According to one of the various embodiments in this invention, this electron-based energy renewal method shows how to isothermally extract environmental heat energy to generate electricity by describing how to make and use an asymmetric function-gated isothermal electron-based power generator such as the asymmetric electron-gated system 1000, illustrated in Figure 4. The system 1000 (Figure 4A) comprises an asymmetric electron-gating function 1003 across a membrane-like barrier space, 1004, that separates two electric conductors 1001 and 1002, which act as a pair of a thermal electron emitter and an electron collector, two electrically conducting leads, 1006 and 1007, connected with each of the electrodes 1001 and 1002 as the two power outlet terminals, which may then be connected with an electrical load 1008. The barrier space 1004 is preferably a special electric insulator that contains no electric conduction materials (i.e., does not conduct electrons through any molecular orbital-associated conduction bands) but allows the thermally emitted electrons to fly ballistically across the emitter and collector.

Therefore, the barrier space 1004 comprises a vacuum space that has no electric conductive materials and/or molecules with molecular orbital-associated electric conduction bands, but allows the thermally emitted electrons to fly through ballistically. The asymmetric electron-gating function, 1003, effectively allows freely emitted thermal electrons, 1005, to fly ballistically, predominantly from the electric conductor (emitter) 1001 through the barrier space, 1004, to the electric conductor (collector) 1002, although the two electric conductors 1001 and 1002 are under the same temperature and pressure conditions. Since the barrier space 1004 is an electrically insulated space without the conventional conductor-based electrical conduction, while still having a unique property that allows thermal electrons to fly through ballistically, it prevents the excess thermal electrons captured by the collector 1002 from conducting back to the emitter except for minimal back emission from the collector that may be controlled by the asymmetric electron-gating function 1003. As a result, the excess thermal electrons captured by the collector 1002 may accumulate and electrostatically distribute themselves, mostly to the collector 1002 electrode surface. Similarly, the excess positive charges (“holes”) left in the emitter may also accumulate and electrostatically distribute themselves, mostly to the emitter 1001 electrode surface. This results in the creation of an electric voltage potential difference across the barrier space 1004 between the emitter electrode 1001 and the collector electrode 1102, in a manner that is analogous to the creation of a transmembrane potential ΔΨ in the protonic bioenergetics systems (Figure 2 and Figure 3).

Note that, in the cases of transmembrane electrostatically localized excess protons (Figure 2 and Figure 3), when a protonic load circuit such as an F_0_F_1_-ATP synthase protonic channel/load is provided, the excess protons typically flow through the ATP synthase protonic channel across the membrane to perform work in driving ATP synthesis (as illustrated in Figure 3). Analogously, when an external electric load circuit is connected between the emitter and the collector, the excess electrons in the collector can flow through the external load circuit back to the emitter. Consequently, in this case, the excess electrons in the collector electrode will pass through an external circuit comprising an electrically conducting lead as an electric outlet 1007 (−) and an electrical load 1008 connected with another wire as electric outlet 1007 (+), and back to the emitter 1001 (Figure 4A). By doing so, a portion of the environmental heat energy associated with the thermal electrons is utilized to perform work through use of the electrical load 1008 in this example. 

Figure 4B illustrates an example of a basic unit of an asymmetric function-gated isothermal electron power generator system 1100 comprising a barrier space 1104, such as a vacuum space that separates a pair of electric conductors 1101 and 1102; one of them has a low-work-function film 1103 surface, and the other has a high-work-function plate 1109 surface. The surface film 1103 is made of a low-work-function material such as Ag-O-Cs, which can have a work function as low as about 0.7 eV, to serve as the emitter. The barrier space 1104 is a special electric insulator space, such as a vacuum space, that does not conduct electricity by the regular electric conduction but allows free thermal electrons 1105 to fly through ballistically. The use of such a barrier space 1104 and low-work-function surface film 1103 enables significant amounts of ambient temperature thermal electrons to be emitted from the film surface into the barrier space 1104 and to fly ballistically towards the collector, which is a high-work-function plate 1109 such as a copper plate that has a work function as high as about 4.6 eV. At ambient environmental temperature around 298 K, such a high-work-function plate 1109, practically has nearly zero emission of thermal electrons from its surface, whereas it can accept the thermal electrons flying through the barrier space from the emitter 1101.

After the thermal electrons 1105 from the emitter 1101 flowing ballistically across the barrier space arrive at the collector 1102, as excess electrons, they as excess electrons will electrostatically repel each other and spread around the electric conductor 1102 (collector) surface in a way quite similar to the behavior of the excess protons in a proton-conductive water body (illustrated in Figure 1c of WO2017/007762 A1 and US 2017/0009357 A1). Similarly, the excess holes (positive charges) left at the emitter will also electrostatically spread around the electrode 1101 (emitter) surface. As a result, this creates a voltage difference between the emitter 1101 and the collector 1102. The use of this voltage difference through the terminals of the electricity outlets 1107 (−) and 1106 (+) can drive an electric current through the load resistance (such as an electric resistor) 1108 to perform electric work, as shown in Figure 4B. This conductive flow of electrons through the external load wire, better known as electricity, is able to continue, as the excess electrons flow conductively through the external circuit back to the emitter, where they will be re-emitted again for the next cycle, and so on, after gaining thermal motion kinetic energy from the environmental heat of the surrounding environment. This explains how the asymmetric function-gated system 1100 is able to isothermally generate electricity by isothermally utilizing heat energy from the environment.

The asymmetric function-gated thermal electron power generator system 1100, as illustrated in Figure 4B, operates isothermally where the temperature at the emitter ($T_{e}$) is equal to that of the collector ($T_{c}$). Under the isothermal operating conditions ($T=T_{e}=T_{c})$, the ideal net flow density (flux) of the emitted electrons 1105 from the emitter 1101 to the collector 1102, which is also defined as the ideal isothermal electron flux ($J_{isoT}$) normal to the surfaces of the emitter and collector (also named as the ideal isothermal electricity current density, defined as amps (A) per square centimeter of the cross-section area of the emitter–collector interelectrode space), can be calculated based on the Richardson-Dushman formulation using the following ideal isothermal current density ($J_{isoT}$) equation:

(9) $$J_{isoT}=AT^{2}(e^{-\left[WF\left(e\right)+e\;\cdot \;V\left(e\right)\right]/kT}\;-\;e^{-\left[WF\left(c\right)+e\;\cdot \;V\left(c\right)\right]/kT})$$

where A is the universal factor (also known as the Richardson-Dushman constant), and can be expressed as $\frac{4\pi mek^{2}}{h^{3}}\approx 120\;Amp/\left(K^{2}.\;cm^{2}\right)$ (where m is the electron mass, e is the electron unit charge, k is the Boltzmann constant and h is Planck constant). T is the absolute temperature in Kelvin (K) for both the emitter and the collector; $WF\left(e\right)$ is the work function of the emitter surface; the term $e\cdot V\left(e\right)$ is the product of the electron unit charge e and the voltage $V\left(e\right)$ at the emitter; k is the Boltzmann constant in the eV/K unit; $WF\left(c\right)$ is the work function of the collector surface; and $e\cdot V\left(c\right)$ is the product of the electron unit charge e and the voltage $V\left(c\right)$ at the collector.

Of particular significance is that the environmental thermal energy can be converted isothermically into electrical power without the need for an external energy-consuming heater or an exhaust, heat sink or the like, so that the energy efficiency is essentially 100%, and is not constrained by the second law of thermodynamics. 

According to one of the various embodiments, when the voltage at the emitter (*V*(*e*)) is zero, such as when the emitter is grounded, the ideal net isothermal electrons flow density across the vacuum space from the emitter 1101 to the collector 1102 can be calculated using the following modified ideal isothermal current density ($J_{isoT\left(gnd\right)}$) equation:

(10) $$J_{isoT\left(gnd\right)}=AT^{2}(e^{-\left[WF\left(e\right)\right]/kT}\;-\;e^{-\left[WF\left(c\right)+e\;\cdot \;V\left(c\right)\right]/kT})$$

**Figure 5:**
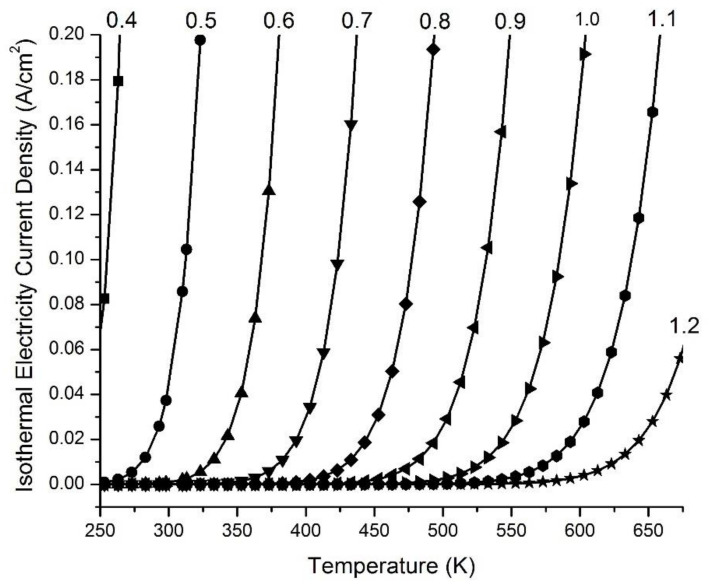
Examples of the isothermal electricity current density (A/cm^2^) curves at an output voltage *V*(*c*) of 3.00 V as a function of operating under environmental temperature *T* for a series of emitters with a low work function of 0.4, 0.5, 0.6, 0.7, 0.8, 0.9, 1.0, 1.1, or 1.2 eV; each of these emitters is grounded and paired with a high-work-function (4.56 eV) collector.

Figure 5 presents examples of the ideal isothermal electricity current density in Amps (A) per cm^2^ (A/cm^2^) at an output voltage *V*(*c*) of 3.00 V as a function of operating environmental temperature *T* with a series of emitter work function (*WF*(*e*)) values including 0.4, 0.5, 0.6, 0.7, 0.8, 0.9, 1.0, 1.1 or 1.2 eV, when paired with the collector work function (*WF*(*c*) = 4.56 eV, copper Cu(110)) and with the emitter grounded. The data indicate that the use of an emitter with a lower work function is highly imperative for isothermally utilizing environmental heat to generate electricity. Therefore, it is a preferred practice to employ an emitter with a low work function, selected from the group consisting of 0.4, 0.5, 0.6, 0.7, 0.8, 0.9, 1.0, 1.1 and 1.2 eV, and/or within a range bounded by any two of these values for the generation of isothermal electricity at a temperature range from 250 K to 673 K.

**Figure 6:**
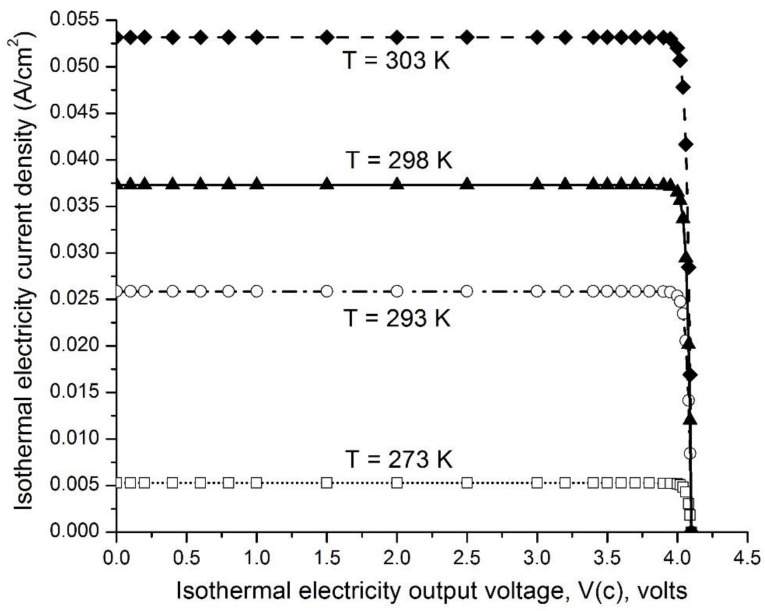
Presents examples of the isothermal electricity current density (A/cm^2^) curves as a function of output voltage *V*(*c*) volts from 0.00 to 4.10 V when operating under an environmental temperature of 273, 293, 298, or 303 K for a pair comprising emitter work function (0.50 eV) and collector work function (4.60 eV), and with the emitter grounded.

Figure 6 presents examples of the ideal isothermal electricity current density (A/cm^2^) curves as a function of output voltage *V*(*c*) from 0.00 to 4.10 V when operating under environmental temperatures of 273, 293, 298, and 303 K for a pair consisting of emitter work function (*WF*(*e*) = 0.50 eV) and collector work function (*WF*(*c*) =4.60 eV, graphene and/or graphite), and with the emitter grounded. These curves indicate that the isothermal electricity current density is pretty much constant (steady) in an output voltage *V*(*c*) range from 0.00 to 4.00 V when operating at each of the environmental temperatures 273, 293, 298, and 303 K. Only when the output voltage *V*(*c*) increases beyond 4.00 V, to the limit of 4.10 V, does the isothermal electricity current density dramatically decrease to zero. The level of the steady-state isothermal electricity current density at an output voltage of 3.50 V increases dramatically with temperature, ranging from 5.26 × 10^−3^ A/cm^2^ at 273 K (0 °C), to 2.59 × 10^−2^ A/cm^2^ at 293 K (20 °C), 3.73 × 10^−2^ A/cm^2^ at 298 K (25 °C), and to 5.32 × 10^−2^ A/cm^2^ at 303 K (30 °C).

These data (Figure 5 and Figure 6) show that significant amounts of isothermal electricity can be generated through an asymmetric function consisting of a low-work-function (e.g., 0.7 eV) electron emitter and a high-work-function (5 eV) electric collector across a barrier space, such as a vacuum space that allows the thermally emitted electrons to fly through ballistically. This enables freely emitted thermal electrons to fly ballistically predominantly from the emitter, through the barrier space, to the electric collector, although the emitter and collector are in the same environmental temperature. Since the barrier space is an electrical insulating space without conventional-conductor-based electrical conduction, but possesses a unique property that allows thermal electrons to fly through ballistically, it prevents excess thermal electrons captured by the collector from being conducting back to the emitter. As a result, this creates a voltage difference between the emitter and the collector. This voltage difference can drive an electric current through a load resistance to perform useful electric work. This electric current is able to continue, as the excess electrons are conducted through the external circuit back to the emitter, where they will be re-emitted again during the next cycle, and so on, after gaining thermal motion kinetic energy from the environmental heat energy. Therefore, this has the potential to power electronic devices, including mobile phones and laptops, forever, which is likely to be transformative in energy science and technologies aiming towards sustainability.

**Figure 7:**
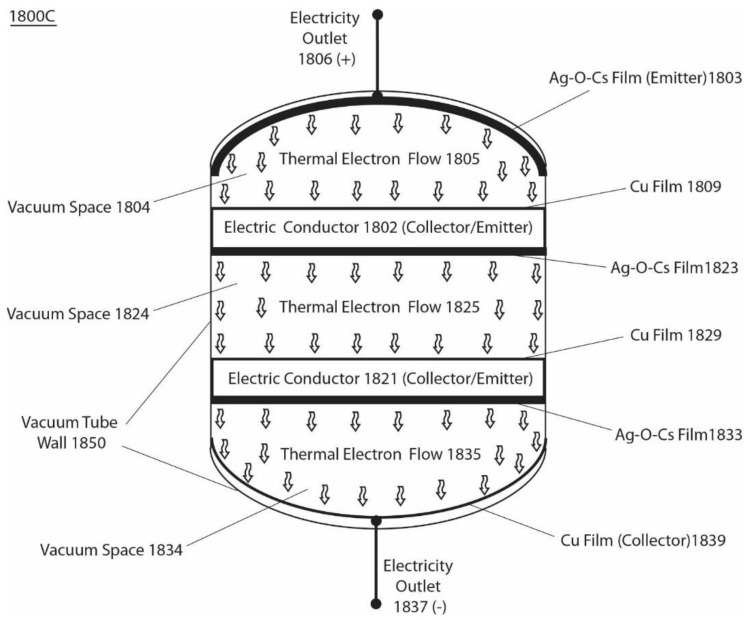
Another example of an integrated isothermal electricity generator system 1800C that has three pairs of low-work-function Ag-O-Cs (0.7 eV) emitters and high-work-function Cu metal (4.56 eV) collectors, operating in series as installed in a vacuum tube container.

Figure 7 presents another example of an integrated isothermal electricity generator system 1800C that has three pairs of emitters and collectors, operating in series, and employing a low work function of Ag-O-Cs (0.7 eV) and a high work function of Cu metal (4.56 eV). The system 1800C (Figure 7) comprises the following components, which are installed in a vacuum tube, from top to bottom: Ag-O-Cs film (emitter) 1803 coated onto the inner surface of dome-shaped top end of the vacuum tube wall 1850 to serve as the first emitter, which has an electricity outlet 1806 (+); a first vacuum space 1804 that allows thermally emitted electrons 1805 to flow through ballistically; a Cu film/plate 1809 to serve as the first collector on the top surface of electric conductor 1802; an Ag-O-Cs film 1823 as the second emitter at the bottom surface of electric conductor 1802; a second vacuum space 1824 that allows thermally emitted electrons 1825 to flow through ballistically; a Cu film/plate 1829 as the second collector on the top surface of the electric conductor 1821; an Ag-O-Cs film 1833 as the third emitter on the bottom surface of the electric conductor 1821; a third vacuum space 1834 allowing thermally emitted electrons 1835 to flow through ballistically; and a Cu film/plate 1839 coated on the inner surface of the inversed-dome-shaped bottom end of the vacuum tube, serving as the terminal collector connected with an electricity outlet 1837 (−). When the isothermal electricity is delivered through the outlet terminals 1806 and 1837 across three pairs of emitters and collectors, the maximum total steady-state operating output voltage is typically about 10.5 V. The total saturation isothermal electricity current density (at an output voltage of 10.5 V) is about 1.55 × 10^−5^ (A/cm^2^) at a standard ambient temperature of 298 K (25 °C) in this example. 

According to one of the various embodiments, any of the isothermal electricity generator systems presented here could be modified for various applications. For example, a typical smart mobile phone device such as an iPhone 12 Pro consumes about 11 Watt-hours per day (24 h). The use of certain isothermal electricity generator systems disclosed in this invention may make it possible to produce a new generation of smart mobile electronic devices that are able to utilize the environmental heat energy from the ambient temperature environment to permanently power the devices without requiring conventional electrical power sources. For instance, the use of an asymmetric function-gated isothermal electricity generator system disclosed here with a chip size of about 40 cm^2^ that has 3 volts (V) of isothermal electricity output of 200 mA as a permanent power source may be sufficient to continuously power a smart mobile phone device forever. That is, based on this invention, isothermal electricity generator systems have the potential to permanently power many electronic devices, including mobile phones and laptops.

Therefore, this energy renewal isothermal electricity invention (WO 2019/136037 A1) has potential in many revolutionary industrial applications, and will likely be transformative in energy science and technologies with respect to providing endless clean energy, supporting sustainable economic development on Earth [30]. More information, including more detailed methods for the creation and use of asymmetric function-gated isothermal electricity generator systems for energy renewal with electrons isothermally utilizing environmental heat energy, is disclosed in the PCT International Patent Application Publication Number WO 2019/136037 A1. 

## 6. Conclusions

On the basis of the work presented here, it is now quite clear that mitochondria are able to isothermally utilize low-grade environmental heat energy (i.e., the thermal motion kinetic energy of transmembrane electrostatically localized protons) associated with the human body temperature of 37 °C to perform useful work, driving the synthesis of ATP. This amazing phenomenon is biophysically enabled through the combination of protonic membrane capacitor formation with the geometric effect of mitochondrial cristae in enhancing the density of localized protons at the cristae tips at which the F_0_F_1_-ATP synthase enzymes are located (Figure 2), and with the asymmetric features of the mitochondrial inner membrane, where the outlets of the redox-driven proton pumps protrude away from the membrane surface to deliver protons into the bulk liquid phase, while the protonic inlet of the ATP synthase is located at the membrane surface (Figure 3). These predicted features have an excellent correspondence with the true structures and functions of mitochondrial cristae [10] and mitochondrial respiratory membrane protein complexes I, II, III, IV, and F_0_F_1_-ATP synthase in terms of their location with respect to the membrane surfaces [75,76,77,80,83,84,85,86,87,88,89,90,91,92]. That is, this finding is well corroborated by the structures and functions of mitochondrial cristae (Figure 2) and is also corroborated by the asymmetric structures of mitochondrial respiratory-coupling sites (Figure 3). 

As shown in Table 1, for an example, with a transmembrane potential of 120 mV, mitochondria obtain as much as −31.7 kJ/mol of local protonic Gibbs free energy $\Delta G_{L}$ from transmembrane electrostatically localized protons utilizing environmental heat (proton thermal motion kinetic energy), which, surprisingly, represents 72% of the total protonic Gibbs free energy $\Delta G_{T}$ (−43.9 kJ/mol), while only 28% is from the classic Mitchellian protonic free energy $\Delta G_{C}$ component (−12.2 kJ/mol). 

Through the newly formulated protonic entropy equation (Equation (7)), it is now, for the first time, clearly demonstrated that, as long as the localized proton concentration $\left[H_{L}^{+}\right]$ is greater than zero, the entropy change ($\Delta S_{L}$) is indeed a negative number for the isothermal environmental heat utilization process in mitochondria. Consequently, we humans, as mitochondria-powered organisms, are not only chemotrophs, but also possess a significant thermotrophic characteristic, isothermally utilizing environmental heat energy from our human body environment to perform work such as ATP synthesis. The entropy change ($\Delta S_{L}$) calculated from Equation (7) for transmembrane electrostatically localized proton-associated isothermal environmental heat utilization was in the range from −95.1 to −107 J/K·mol, for which it is now, for the first time, numerically demonstrated that the transmembrane electrostatic proton localization, including the formation of the protonic membrane capacitor (Figure 2 and Figure 3), does indeed represent a negative entropy event (Table 1). This also explains the relationship between the local protonic negative entropy change ($\Delta S_{L}$) and the mitochondrial environmental temperature (*T*) and the local protonic Gibbs free energy ($\Delta G_{L}=T\Delta S_{L}$) in the utilization of isothermal environmental heat. 

This thermotrophic function is able to lock a significant fraction of the environmental heat energy into ATP chemical energy. The energy efficiency for the utilization of total protonic Gibbs free energy ($\Delta G_{T}$ including $\Delta G_{L}$) in driving the synthesis of ATP is estimated to be about 60%, indicating that a significant fraction of the environmental heat energy associated with the thermal motion kinetics energy (*k_B_T*) of transmembrane electrostatically localized protons was locked into the chemical form of energy in ATP molecules. Therefore, mitochondria are indeed able to isothermally utilize the environmental heat energy through electrostatically localized protons to help drive the synthesis of ATP, a significant thermotrophic feature with profound scientific implications.

Proton-coupling bioenergetics systems operate widely, and are present in nearly all organisms known today. Through the present study, it is now quite clear that this special thermotrophic process, which is associated with transmembrane electrostatically localized protons, has probably already been occurring for billions of years on Earth. Therefore, there are two thermodynamically distinct types (A and B) of energy processes naturally occurring on Earth, based on their properties with respect to whether they follow the second law of thermodynamics or not. Type-A includes energetic processes such as glycolysis, tricarboxylic acid cycle, redox-driven electron transport, and many of the chemical reactions and processes in our test tubes, computers, and cars, which apparently well follow the second law; Type-B energetic processes, represented by the thermotrophic function (Table 1 and Figure 1, Figure 2 and Figure 3), here, do not necessarily have to be constrained by the second law, owing to the special asymmetric function. That is, the second law still remains a very good law. However, it does not necessarily always have to be universal, as also implied by several independent studies [3,11,22,23,24,25,26,27,28,29,30,31,32].

The discovery of the thermotrophic function isothermally utilizing environmental heat energy, reported here, may have profound scientific and practical implications in bettering our fundamental understanding of bioenergetics and energy renewal [11] for the purpose of sustainable development on Earth. With the new knowledge learned from this discovery, it may be possible to obtain benefits from mimicking this biophysical molecular-scale process in order to create a new way [11,12,30] of producing useful energy by isothermally utilizing environmental heat energy from the ambient environment. 

Inspired by the discovery of the protonic thermotrophic function, a new invention (WO 2019/136037 A1) [30] was developed for energy renewal on the basis of isothermal environmental heat energy utilization with an asymmetric electron-gated function system to generate electricity, which, to a certain extent, mimics Type B energy processes.

As highlighted above, a basic asymmetric function-gated isothermal electron power generator system (Figure 4B) comprises a barrier space, such as a vacuum space, that separates a pair of electric conductors, one of which has a low-work-function film surface and the other of which is a high-work-function plate. The surface film is made of a low-work-function material such as Ag-O-Cs, which has a work function as low as approximately 0.7 eV, and serves as the emitter. The barrier space is a vacuum space that does not conduct electricity through regular electric conduction, but allows free thermal electrons to fly through ballistically. The use of such a barrier space and low-work-function surface film enables significant amounts of ambient temperature thermal electrons to be emitted from the film surface into the barrier space and to fly ballistically towards the collector, which is a high-work-function plate, such as a copper plate, which can have a work function as high as approximately 4.6 eV. Practically, at ambient temperatures of around 298 K, such high-work-function plates have nearly zero emissions of thermal electrons from their surface, while being able to accept the thermal electrons flying through the barrier space from the emitter. This enables freely emitted thermal electrons to predominantly fly ballistically from the emitter through the barrier space to the electric collector, despite the emitter and collector being at the same environmental temperature. After the thermal electrons from the emitter flowing ballistically across the barrier space arrive at the collector, as excess electrons, they electrostatically repel each other and spread around the electric conductor (collector) surface, analogously to the behavior of excess protons in a protonic conductor in the thermotropic function (Figure 2 and Figure 3). Similarly, the excess holes (positive charges) left at the emitter will also electrostatically spread around the electrode (emitter) surface. As a result, this creates a voltage difference between the emitter and the collector. The use of this voltage difference through the terminals of electricity outlets can drive electric current through load resistance in order to perform electric work, as shown in Figure 4B. This conductive flow of electrons through the external load wire, better known as electricity, is able to take place continuously, as the excess electrons flow conductively through the external circuit back to the emitter, where they will subsequently be re-emitted again during the next cycle, and so on, after gaining thermal motion kinetic energy from the environmental heat of the surrounding environment. This explains how the asymmetric function-gated isothermal electron power generator system is able to isothermally generate electricity by isothermally utilizing heat energy from the environment.

The asymmetric function-gated isothermal electricity generator systems have the potential to generate substantial amounts of electricity (data shown in Figure 5 and Figure 6) to power many electronic devices, including mobile phones and laptops, forever. Thus, this invention has the potential for many industrial applications, and is likely to be transformative in energy science and technologies aiming towards sustainability on Earth. Its continuous forever clean energy renewal function (Figure 4, Figure 5, Figure 6 and Figure 7), mimicking the energy Type-B processes in isothermally utilizing endless environmental heat energy, could help to liberate all peoples from their dependence of fossil fuel energy, thus helping to reduce greenhouse gas CO_2_ emissions and control climate change. Therefore, the author hereby encourages both public and private supports to accelerate the R&D efforts on isothermal environmental heat energy utilization at national and/or international scales, to provide the envisioned continuous clean energy for all peoples, with the aim of a shared common future for humanity on the planet. 

## Figures and Tables

**Table 1:** Mitochondrial protonic energetics features and associated properties including the local protonic entropy change ($\Delta S_{L}$ ), the classic protonic Gibbs free energy ($\Delta G_{C}$), the local protonic Gibbs free energy ($\Delta G_{L}$) and the total protonic Gibbs free energy ($\Delta G_{T}$), calculated as a function of membrane potential ΔΨ using Equations (1)–(7) based on the measured properties ($pH_{pB}$, $pH_{nB},$ ΔΨ) with the known reaction medium compositions of Ref. [52]. The cation concentrations, proton–cation exchange equilibrium constants and cation exchange reduction factor (1.29) are from Refs. [8,10]; and the temperature *T* = 310 K. Adapted and updated in part from Ref. [8].

ΔΨ (mV)	$pH_{pB}$	$pH_{nB}$	$\left[H_{L}^{+}\right]$ (mM)	$\Delta S_{L}$ J/K·mol	$\Delta G_{C}$ (kJ/mol)	$\Delta G_{L}$ (kJ/mol)	$\Delta G_{T}$ (kJ/mol)	$\Delta G_{Syn}$ (kJ/mol)	$\Delta G_{Chem}$ (kJ/mol)
50	7.25	7.35	5.30	−95.1	−5.42	−29.5	−34.9	−24.5	−22.0
55	7.25	7.35	5.83	−95.9	−5.90	−29.7	−35.6	−24.5	−22.0
60	7.25	7.35	6.36	−96.6	−6.38	−30.0	−36.3	−24.5	−22.0
65	7.25	7.35	6.89	−97.3	−6.86	−30.2	−37.0	−24.5	−22.0
70	7.25	7.35	7.42	−97.9	−7.35	−30.4	−37.7	−24.5	−22.0
75	7.25	7.35	7.95	−98.5	−7.83	−30.5	−38.4	−24.5	−22.0
80	7.25	7.35	8.48	−99.0	−8.31	−30.7	−39.0	−24.5	−22.0
90	7.25	7.35	9.55	−100	−9.28	−31.0	−40.3	−24.5	−22.0
100	7.25	7.35	10.6	−101	−10.2	−31.3	−41.5	−24.5	−22.0
110	7.25	7.35	11.7	−102	−11.2	−31.5	−42.7	−24.5	−22.0
120	7.25	7.35	12.7	−102	−12.2	−31.7	−43.9	−24.5	−22.0
130	7.25	7.35	13.8	−103	−13.1	−31.9	−45.1	−24.5	−22.0
140	7.25	7.35	14.8	−104	−14.1	−32.1	−46.2	−24.5	−22.0
150	7.25	7.35	15.9	−104	−15.1	−32.3	−47.4	−24.5	−22.0
160	7.25	7.35	17.0	−105	−16.0	−32.5	−48.5	−24.5	−22.0
170	7.25	7.35	18.0	−105	−17.0	−32.6	−49.6	−24.5	−22.0
180	7.25	7.35	19.1	−106	−18.0	−32.8	−50.7	−24.5	−22.0
190	7.25	7.35	20.2	−106	−18.9	−32.9	−51.9	−24.5	−22.0
200	7.25	7.35	21.2	−107	−19.9	−33.1	−52.9	−24.5	−22.0

## Data Availability

The data presented in this study are available in article here.
